# Atoms, bits, and cells^☆^

**DOI:** 10.1016/j.atg.2015.07.004

**Published:** 2015-08-04

**Authors:** David Glazer

**Affiliations:** Google Inc.

Nicholas Negroponte, in his 1995 book *Being Digital*, popularized the notion of the worlds of Atoms and Bits. The history of civilization to date was largely fueled by advances in the world of Atoms, with the 19th-century Industrial Revolution marking a notable acceleration:

The Industrial age, very much an age of atoms, gave us the concept of mass production, with the economies that come from manufacturing with uniform and repetitious methods in any one given space and time.

We were at the beginning of a similar acceleration in the world of Bits, with an information revolution marked by Tim Berners-Lee's invention of the web browser in 1990. The free flow of information led to a new kind of scale:

In the information age, mass media got bigger and smaller at the same time. In the post-information age, we often have an audience the size of one. Everything is made to order, and information is extremely personalized.

The world of Bits is continuing to transform the world of Atoms. Services like Amazon, AirBnB, and Uber show the potential for organizing information from many sources, and using it to deliver tailored real-world experiences to just the right person at just the right time.

Today we're at the beginning of a third revolution, as the world of Cells enters the world of Bits. New measurement tools, including high-throughput genomic sequencing, single-cell analysis (Macosko et al., 2015), and mobile sensors (Otis and Parviz, 2014) are giving researchers and clinicians access to orders of magnitude more data than they ever had before. Life science is moving from the era of artisan-scale observation to the era of factory-scale analysis. The great acceleration of progress we've seen in other fields is now coming to healthcare.

At Google, we have more than 15 years of experience with finding the value in information. We have built tools to organize information and make it accessible and useful to people everywhere. Our global infrastructure, which powers Search, Maps, YouTube, and more, is available to everyone via the Google Cloud Platform. Google Genomics is working to bring similar tools to the world of Cells, and provide scalable services to scientists, clinicians, and patients.

In this article, we look at how the combination of data science and life science, or the worlds of Bits and Cells, could fundamentally change how patients are treated and how discoveries are made. With so many researchers working hard on the challenge, we believe that precision medicine is within our reach.

## 1 The vision

Cheap data production and storage have enabled many fields to make the leap from qualitative to quantitative, and from one-size-fits-all to precision value delivery. Consider weather forecasts: as satellites and sensors around the world collect massive amounts of information, computers have analyzed these data troves to make sense of all sorts of weather phenomena, resulting in more reliable information on what tomorrow's weather may bring. Similarly, we witnessed a sea change in our ability to get around as geographic information became easier to obtain. Big data has made it possible not only to build accurate maps of virtually any place on the planet, but also to provide precision navigation that uses real-time traffic information to automatically route you around congestion or road construction.

Amazon uses information technology to allow every reader to find just the books (and other products) they want. Companies like AirBnB and Uber are managing information from global networks of service providers (property owners, drivers) to give global consumers more flexibility than was possible with last century's technology. These new precision services are benefiting all users of retail, lodging, and transportation — that is, all of us.

These and other industries have transformed themselves by using large-scale computational resources and high-powered analytical tools to unlock the value in data. Doing the same in biology will lead to new interventions based on new understanding of the genetic mechanisms of disease progression and resilience; early warning of impending disease using individual health data gathered via wearable sensors; access to knowledge in the untapped troves of clinical data; and treatments chosen by how each patient best responds to each method of prevention and therapy.

This transformation will be accelerated as we find ways to free data now trapped in silos, contributing to a globally accessible resource that will allow scientists to query not just the data they've generated, but also data generated by global collaborators and in publicly available databases. Looking at common diseases or traits in studies requiring hundreds of thousands or millions of people for statistical significance will no longer be an insurmountable challenge.

**Fig. 1:**
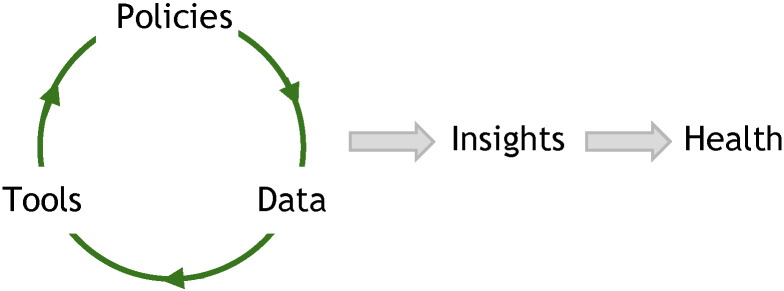
Building a virtuous cycle.

Realizing this vision will take organization of large amounts of data, scalable processing and analysis tools, and updated policies to support the new possibilities (Fig. 1).

## 2 Driven by data

Remarkable improvements in the cost of DNA sequencing have removed many rate-limiting factors in genomics, shifting the bottleneck from generating data to interpreting and using that data. Just 15 years ago, the global scientific community invested significant time and funding to produce a single human genome sequence; today, the highest-throughput systems can generate sequence data for nearly 50 genomes per day. In 2013, scientists estimated that the world's sequencing capacity could generate 15,000 terabytes of compressed genetic data per year, and that capacity was increasing three- to five-fold annually (Schatz and Langmead, 2013).

Cheap sequencing has made large-scale studies possible, and more are launching all the time. The Autism Speaks MSSNG Project aims to sequence 10,000 samples from families with autism, leading to what is expected to be the world's largest database of autism-related sequencing data. In the UK, the 100,000 Genomes Project is working to sequence 100,000 people, with early goals of shedding light on rare diseases and common cancers. 23andMe recently announced that it had genotyped its millionth customer (Wojcicki, 2015). Other examples include the VA's Million Veteran Program and the Precision Medicine Initiative announced by President Obama in the 2015 State of the Union address.

## 3 A transformation in tools

**Fig. 2:**
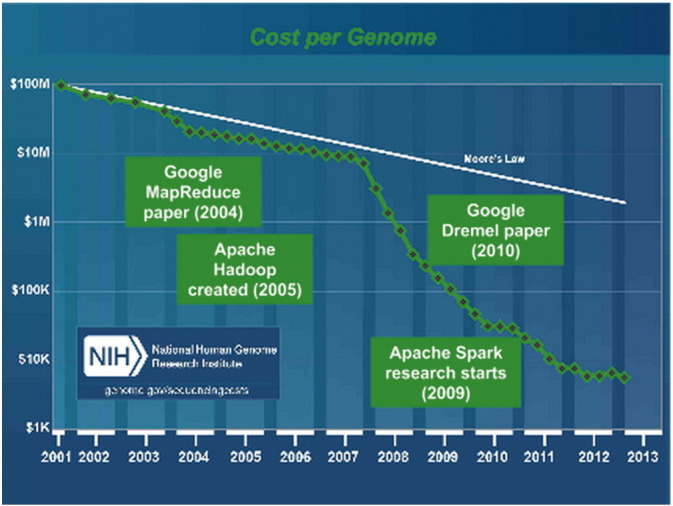
Data science milestones.

Having a lot of data is a necessary first step, but is not sufficient — we also need a shift in how we process and interact with biological data. The knowledge we want to mine from sequencing data will depend on algorithms that can spot patterns across multiple datasets, find the quietest signals in the noisiest data, and teach themselves to answer questions better as they crunch more and more information. Fortunately, the world of data science has been building the tools we need (Fig. 2).

MapReduce, originally built at Google to regenerate the web index for better accuracy and speed of Search results (Dean and Ghemawat, 2004), was designed to take advantage of many thousands of processors, each handling one slice of a task. Since then, the MapReduce programming model has given birth to a family of tools, including Hadoop, Spark, Flume, Millwheel, and Dataflow. In our tests with data from the 1000 Genomes Project, we showed that MapReduce-like tools work well on genomic data — in one example, sorting samples by ancestry using principal component analysis.

Dremel, originally built at Google for interactive analysis of trillion-row tables (Melnik et al., 2010), uses thousands of disk drives, each holding a slice of data. By using Google BigQuery, a publicly available Dremel-based service, we are able to parse terabytes of genomic variant data and provide answers in seconds. This tool can be used to quickly detect patterns between genetic variants and disease, populations, epidemiology, and more.

In *The Unreasonable Effectiveness of Data* (Halevy et al., 2009), we learn that in domains where enough data is available, machine intelligence can be extremely effective at finding patterns and insights, with initial successes in language translation and speech recognition. Work in the field has continued, with new techniques being developed and applied to a variety of problem areas.

**Fig. 3:**
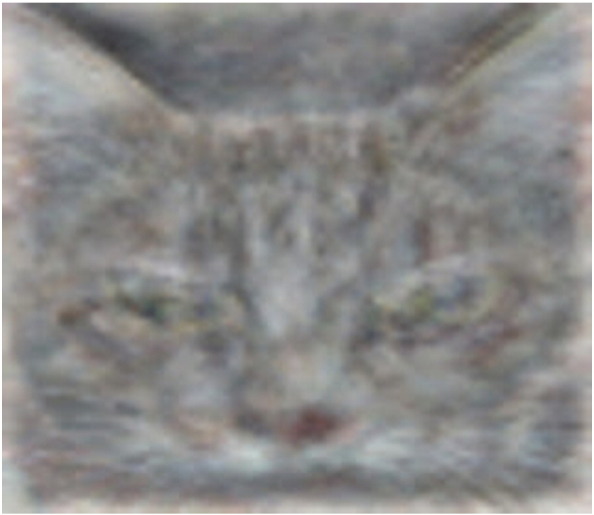
A commonly found object in YouTube videos.

Deep learning, an approach to automatically finding the high-level abstractions beneath the surface of low-level data, is one approach that has promise for biological datasets. In 2012, we reported (Le et al., 2012) on using deep learning to detect common objects in 10 million YouTube videos. Although the computer wasn't told what to look for, analysis across so many images allowed the algorithms to find patterns. For example, our experiment yielded a composite image of a cat (Fig. 3); having seen this particular pattern of pixels in so many images, computers were able to determine that there was something important about a cat (at least to the YouTube community). Feeding massive genomic and phenotypic datasets into these machine-learning algorithms could result in pattern detection in the areas of disease progression, the sequence of genetic variant additions that lead to cancer, gene expression, and more.

**Fig. 4:**
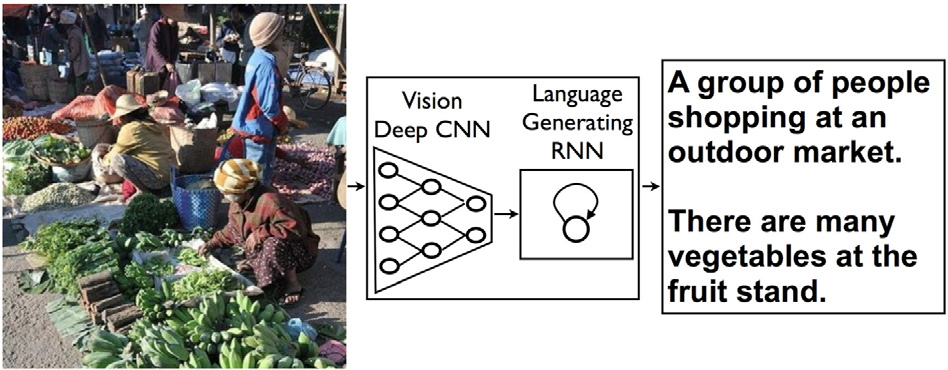
Generating captions by translating from pictures to text.

The success of IBM's Watson on Jeopardy! in 2011 was based on years of machine learning geared to the game show's format. Machine learning was used in 2014 for auto-captioning, essentially translating from the language of images into English (Vinyals et al., 2014) (Fig. 4). Earlier this year, Google reported on how our DeepMind technology taught itself to play Atari video games (and win!) (Mnih et al., 2015). The system was able to play at least as well as a professional human game tester across a set of 49 games, without having any knowledge of the rules of any of the games.

These examples are not just laboratory experiments — the tools that make them possible are available today, and the results are in daily use by many millions of users of speech recognition, language translation, and more. As experts in data science and life science work together, and as enough data becomes available to analyze, these algorithms could teach themselves to auto-annotate patient charts, suggest diagnoses, spot links between genotype and phenotype, and even recommend treatment options most likely to succeed for an individual patient.

## 4 Policies and paradigms

Realizing the vision of precision medicine requires more than just data and tools. We'll need fruitful collaborations among academia and industry, including people steeped in life sciences as well as experts from other fields who can offer solutions that aren't traditionally used in biology or medicine. Enabling these collaborations requires updates to the ways we work together.

In the world of technology, it's long been known that vibrant ecosystems arise from interoperable tools, connected by standard programming interfaces, known as APIs. For example, one reason the Web has grown as fast as it has is that any web browser (e.g. Chrome, Safari) can work with any website, so that the providers of information and consumers of information can evolve separately. Bringing similar interoperability to the world of life science information can have similar benefits.

**Fig. 5:**
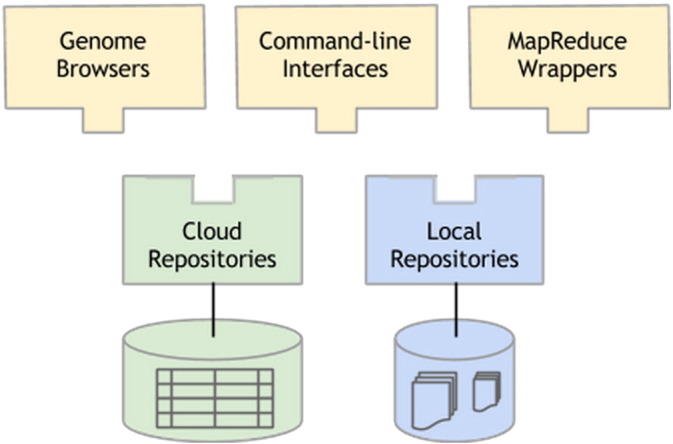
Interoperability: one API, many apps.

The Global Alliance for Genomics and Health (GA4GH, genomicsandhealth.org) is a global partnership with the mission of establishing standards and policies that allow scaling and responsible sharing of life science data. The GA4GH has developed a web-based API, implemented by EBI, Google, and many others, that allows users to import, process, manage, and analyze genomic data at scale. The API enables free and open exchange of data across institutions to facilitate collaborative research and analysis; it also lets users build their own tools and use tools from others (Fig. 5). Any tool that calls the API can work with any data repository that exposes the API. We hope this API will allow scientists to ask bigger questions, and the interoperability will allow all of us to benefit from each other's work.

## 5 Policy progress

Technical policies like those discussed above remove some barriers to progress. There are other potential pitfalls that could make it more challenging to achieve these grand goals. Current practices have evolved for good reason, and take time to change to reflect the new potential benefits (and risks) of today's tools.

One recent example is the NIH policy on the use of cloud computing to store and analyze controlled-access dbGaP data. When the dbGaP policies were originally written, public cloud providers didn't exist, and the internet itself was relatively new. As a result, the policies didn't provide clear guidance around the use of the cloud, leading to a 2014 ban on cloud use until the policies could be clarified. The guidance was updated in March of 2015 — investigators may now request permission to use cloud computing services when submitting a dbGaP Project Request, as long as they show that they are exercising appropriate care in how they use those services (NIH, 2015). Policies had lagged behind tools and become a bottleneck; they have now caught up.

Another example is the evolution of consent. Today's technology makes it easy for data collected by one organization to be securely made available for analysis by many qualified researchers in many locations. That ‘collect once, analyze often’ democratization of access is a value multiplier for data. Unfortunately, many of today's consent forms were written in a world of siloed research, without anticipation of widespread sharing. New studies (such as the MSSNG project, Autism Speaks, 2015) and new work on the legal front (such as Sage's Portable Legal Consent framework, Wilbanks, 2012) are helping policies catch up with technology.

## 6 Going forward

The worlds of Atoms, Bits, and Cells are coming together to the benefit of all of us. We need ongoing collaboration from leaders in all fields to collect more data, build better tools, and thoughtfully upgrade our policies. Google Genomics is part of a growing movement to bring progress from the last 20 years of data science to the world of life science. Together, we can unlock the value in our information, and make the vision of precision medicine real. It is within our reach to fully transform medicine — that very possibility gives us a responsibility to achieve it.

## References

[bb0005] Autism Speaks (2015). MSSNG: Changing the Future of Autism with Open Science. https://www.mss.ng/researchers.

[bb0010] Dean J., Ghemawat S. (2004). MapReduce: simplified data processing on large clusters. OSDI'04: Sixth Symposium on Operating System Design and Implementation, San Francisco, CA.

[bb0015] Halevy A., Norvig P., Pereira F. (2009). The Unreasonable Effectiveness of Data. Google 1541-1672/09 ©.

[bb0020] Le Q.V., Ranzato M.’.A., Monga R., Devin M., Chen K., Corrado G.S., Dean J., Ng A.Y. (2012). Building high-level features using large scale unsupervised learning. Proceedings of the 29th International Conference on Machine Learning, Edinburgh, Scotland, UK.

[bb0025] Macosko E.Z., Basu A., Satija R., Nemesh J., Shekhar K., Goldman M., Tirosh I., Bialas A.R., Kamitaki N., Martersteck E.M., Trombetta J.J., Weitz D.A., Sanes J.R., Shalek A.K., Regev A., SA M.C. (2015). Highly parallel genome-wide expression profiling of individual cells using nanoliter droplets. Cell.

[bb0030] Melnik S., Gubarev A., Long J.J., Romer G., Shivakumar S., Tolton M., Vassilakis T. (2010). Dremel: interactive analysis of web-scale datasets. Proc. of the 36th Int'l Conf on Very Large Data Bases.

[bb0035] Mnih V., Kavukcuoglu K., Silver D., Rusu A.A., Veness J., Bellemare M.G., Graves A., Riedmiller M., Fidjeland A.K., Ostrovski G., Petersen S., Beattie C., Sadik A., Antonoglou I., King H., Kumaran D., Wierstra D., Legg S., Hassabis D. (2015). Human-level control through deep reinforcement learning. Nature.

[bb0040] NIH (2015). NIH Security Best Practices for Controlled-Access Data Subject to the NIH Genomic Data Sharing (GDS) Policy. http://www.ncbi.nlm.nih.gov/projects/gap/pdf/dbgap_2b_security_procedures.pdf.

[bb0045] Otis B., Parviz B. (2014). Introducing Our Smart Contact Lens Project. http://googleblog.blogspot.com/2014/01/introducing-our-smart-contact-lens.html.

[bb0050] Schatz M.C., Langmead B. (2013). The DNA data deluge. IEEE Spectr..

[bb0055] Vinyals O., Toshev A., Bengio S., Erhan D. (2014). A picture is worth a thousand (coherent) words: building a natural description of images. http://googleresearch.blogspot.com/2014/11/a-picture-is-worth-thousand-coherent.html.

[bb0060] Wilbanks J.T. (2012). Portable Legal Consent Overview. http://sagecongress.org/WP/wp-content/uploads/2012/04/PortableLegalConsentOverview.pdf.

[bb0065] Wojcicki A. (2015). Power of One Million. http://blog.23andme.com/news/one-in-a-million.

